# Eight-Year Investigation of the Impact of the Clerkship Administrator Certificate Program

**DOI:** 10.7759/cureus.24024

**Published:** 2022-04-11

**Authors:** Alison D Ricker, Donnita Pelser, Gary L Beck Dallaghan

**Affiliations:** 1 Office of Medical Education, Geisel School of Medicine at Dartmouth College, Lebanon, USA; 2 Pediatrics, University of Kansas School of Medicine Wichita, Wichita, USA; 3 Office of Medical Education, University of North Carolina at Chapel Hill School of Medicine, Chapel Hill, USA

**Keywords:** realistic evaluation, quality improvement, professional development, clerkship coordinator, medical student education

## Abstract

Introduction: The roles and responsibilities of the administrative staff supporting required clinical medical student experiences have evolved. In 2004, the Association of American Medical Colleges Central Group on Educational Affairs (CGEA) offered for the first time the Clerkship Administrator Certificate Program. This program requires the completion of a series of workshops and a project and results in a certificate. Research related to long-term outcomes of professional development programs such as this is limited. The purpose of our study was to explore the impact this professional development program had on the careers of participants.

Methods: We conducted a survey of those who completed the qualifying workshops from 2010 to 2018. The survey was based on program content, including questions to explore the self-described impact on their careers. Categorical and scaled data were summarized using descriptive statistics. The realistic evaluation framework was used to guide inductive and deductive content analysis, allowing respondent interpretations and context to define outcomes.

Results: Out of 244 invitations, 50 (20.5%) responded. Of the respondents, 40 still work in medical education. Scaled responses (strongly disagree to strongly agree) were positive. The individual's motivation, departmental climate, and other contextual factors (experience, collaborators, time) impacted workshop participants’ ability to complete the certificate program. Those who completed certification noted various forms of recognition locally, ranging from special recognition by the chair, raises, and promotions. Additionally, participants felt more confident and accomplished in their careers.

Conclusions: Although positively rated, the success of this program had differential outcomes depending on participant contexts. Unintended results for participants completing the program resulted in promotions. Applying the realistic evaluation framework provided insights to improve the program.

## Introduction

For the past 20 years, the role of the administrative staff in supporting the required clinical medical student experiences has evolved [1]. The fifth edition of The Guidebook for Clerkship Directors offers suggestions on how clerkship administrators complement the educational mission in ways never before considered [2]. Accreditation requirements and evolving medical school curricula necessitate administrative staff to invest in the clerkship administrator role as a career [3-4].

To offer professional development for clerkship administrators, the Central Group on Educational Affairs (CGEA) of the Association of American Medical Colleges began offering a certificate program in 2003. The CGEA Clerkship Administrator Certificate Program (CACP) was delivered for the first time in 2004. The CGEA is an organization for medical educators from all disciplines as well as across the continuum of medical education. A series of three workshops are presented in one setting. Since then the program has been delivered regularly at the CGEA as well as at the Council on Medical Student Education in Pediatrics (COMSEP), Association of Directors of Medical Student Educators in Psychiatry (ADMSEP), and multiple medical schools. We recently published an overview of this program [5].

Programs such as CACP are of value to both participants and institutions due to their structure. Planners developed the program to ensure core content was completed in a four-hour session, which minimized travel expenses and time away for clerkship administrators. Although there have been many individuals who have participated in the program, it is still unclear what impact the program has had on participants. 

Part of the challenge in studying programs such as CACP stems from understanding the individual motivations, departmental cultures, and contextualized social situations of the participants. We, therefore, sought to explore the impact of CACP using a survey to obtain both quantitative and qualitative responses. The richness of responses led us to apply Pawson and Tilley’s realistic evaluation framework [6] to better understand the findings.

The realistic evaluation seeks to identify what works for whom in what circumstances [6-8]. The framework posits that outcomes (O) are a result of context (C) and mechanisms (M). Pawson and Tilley [6] conceptualized it as C + M = O. Contexts are defined as the conditions under which mechanisms operate. Mechanisms can be thought of as forces, interactions, or feedback processes operating within contexts. Further application of the original configuration by Pawson and Tilley suggested that the combination of context and mechanism is not a linear function. Instead, Westhorp suggested that outcomes are a function of contexts and mechanisms [O=fx(CM)] [9]. This distinction supports the notion that multiple mechanisms may be acting within a context resulting in different outcomes. Realistic evaluation allows program planners to, therefore, develop theories about programmatic outcomes, which can then be tested using observational hypotheses.

The purpose of this project was two-fold. We first wanted to obtain long-term feedback on aspects of the certificate program content. For this, we asked specific questions related to the content of the certificate program. Second, we wanted to determine how to improve content and expectations for future participants using the realistic evaluation framework. We used open-ended questions on the survey to construct these findings.

## Materials and methods

Sample

All individuals who participated in the workshops for the CACP from 2010 to 2018 were invited to complete the survey. Although we had contact information from when individuals completed the program, we verified email addresses by searching institutional websites or internet search engines.

Survey

The survey evaluated the CACP content. Additional questions were asked related to the perceived impact of the program, both personally and professionally. The authors developed the questions that included Likert scales and open-ended questions. We invited input from colleagues to read the survey for clarity, making edits to questions where recommended. The survey had 13 questions and is available as a supplemental electronic file. 

The survey was created using QualtricsTM (Seattle, WA). The anonymous link was sent with two reminders at week two and week four after the initial invitation.

The University of North Carolina Institutional Review Board reviewed and approved this study as an exempt (UNC IRB No. 20-0846).

Analysis

Categorical and scaled items on the survey were summarized using descriptive statistics. Summaries were conducted using IBM SPSS, Inc. v. 26 (IBM Inc., Armonk, NY).

The realistic evaluation framework was used in our study in an interpretivist fashion [6]. Crafting meaningful contexts, mechanisms, and outcomes (CMO) necessitates intimate familiarity with the subject under investigation [10]. Two of the authors (DP, GLBD) provide a deeper understanding of the program because they have developed and refined content for this program since its inception in 2004. Narrative comments were analyzed by applying inductive thematic analysis of the narratives by GLBD, which was then reviewed by the other authors. We met virtually and through email exchanges to construct meaning from the themes. These were then aligned with the realistic evaluation framework. Results of the thematic analysis, as well as quantitative survey responses, formed the basis for applying realistic evaluation, allowing us to characterize the complexities of characterizing outcomes constructed for the CACP. 

## Results

Participants attending the Clerkship Administrator Certificate Program workshops from 2010 to 2018 (n=249) were eligible to complete the survey. Of those, two were deceased and two did not have verifiable email addresses, leaving a total sample of 245 participants. We received 50 (20.5%) completed surveys.

Of the respondents, 45 (90%) continue to work in medical education in some capacity. Participants hailed from many disciplines or departments when they completed the workshops. However, pediatrics was represented more than all other disciplines, followed distantly by psychiatry (Table 1). The greater numbers from pediatrics reflect the regular presentation of this program at the COMSEP annual meeting.

**Table 1:** Number of respondents by department. OB/GYN, obstetrics/gynecology

Department	Number
Emergency medicine	1
Family and community medicine	3
Internal medicine	5
Office of medical education	4
Neurology	2
OB/GYN	1
Pediatrics	24
Psychiatry	9
Surgery	1

Thirty-six (72%) respondents completed the program requirements for certification. Of these, 33 applied their project to the clerkship and 26 undertook additional projects. Twelve submitted their project as a poster presentation at a regional or national meeting. Three submitted their final project to a peer-review journal.

**Figure 1:**
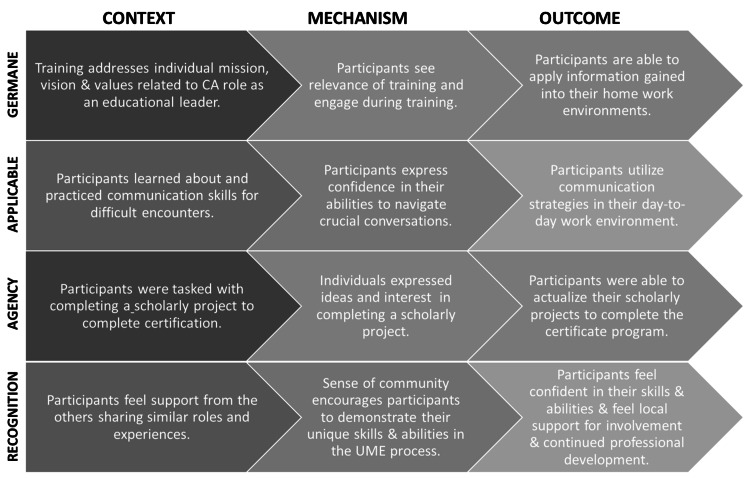
Realistic evaluation analysis results. Context, mechanisms, and outcomes (CMOs) were identified based on survey responses. These CMOs are now being used to guide data collection processes to evaluate future CACP sessions. CMOs, context, mechanisms, and outcomes; CACP, Clerkship Administrator Certificate Program

A set of CMOs developed by the authors is detailed below and summarized in Figure 1. The CMO configurations are hypotheses about the anticipated outcomes of a program. We will explore the four we hypothesized, addressing them based on scaled and narrative comments. The four configurations we identified include: germane, applicable, agency, and recognition.

The first CMO labeled “germane” was built on the premise that clerkship administrators recognize their role as a leader in medical education. The activities in the workshops (e.g., goal setting, sensitive communication skills) provided the mechanisms for participants to apply during the program. We hypothesized the outcome of "germane" context and mechanisms would be applied in their work. Based on the level of agreement from the survey (Table 2), 44 (88%) strongly agreed they recognize their leadership abilities in medical education.

**Table 2:** Responses to program content. Numbers represent the total number of responses to scaled items.

	Strongly disagree/disagree	Somewhat disagree	Neutral	Somewhat agree	Strongly agree/agree
I know what my values are in relation to my work	1	1	1	2	45
I recognize personality types better having completed the Myers-Briggs Test	2	0	6	8	34
I know the strengths I bring to my work	0	1	0	0	49
I recognize my areas for professional growth.	1	0	0	5	44
I am confident in my leadership abilities and how they fit into my job	0	0	2	4	44

“The program provided me with: (i) useful insight for best practices in managing clerkships, (ii) an opportunity to network and learn from others and (iii) an opportunity to conduct some research in the area.” - *Medical Student Education*

“It strengthened my confidence in my role and allowed me to be a stronger voice on campus, which has resulted in my being asked to join committees and a task force representing all clerkship coordinators on campus.” - *Family Medicine*

The goal of every professional development program is that participants find utility in the sessions, which was labeled “applicable.” Gaining specific communication skills to address challenging situations is something clerkship administrators face daily. The activities in this workshop allowed participants to practice skills using a variety of case scenarios. The outcome was their ability to approach challenging conversations with more confidence. The following quote is indicative of others about this particular CMO.

“I feel that the program really gave me the skills to address difficult situations and find the best way to approach people that I may disagree with or have different personality types. This goes for both personal and professional life.” - *Psychiatry*

“This program helped me in my current role as an Administrative and HR Manager. I can better assess personalities and gained knowledge and tools to better help my team move forward and have continued success.” - Psychiatry

The requirement for participants to complete a project assumed participants had baseline skills to complete a quality improvement initiative. During the program, examples of projects previous participants had completed were explained to participants. They were also encouraged to partner with their director to obtain mentoring to facilitate the successful completion of a project. During the sessions, ideas were generated and participants seemed motivated to initiate their projects. Thus, our anticipated outcome was that participants felt a sense of “agency” and that they would complete the project and thereby receive their certificate. However, results were mixed based on the survey. For some, they had no trouble completing the project requirement.

“It was actually fun to come up with a project. I have millions of ideas all the time for student education research.”- *OB/GYN*

“The project required by the program challenged me to "dig deeper" and view the clerkship from new angles.”- *Family Medicine*

Completing a project was required to have completed the program and receive a certificate. For some respondents, completing the project seemed to be of low importance or that current work duties were too time-consuming.

“Carving out time to develop the project. I am both the Student Coordinator and Medical Education Office for our Children's hospital. Currently working with approximately 500 student rotations per academic year.” - *Pediatrics*

Finally, the goal orientation for participating in the program was unknown. Although attending the workshops gave a sense of recognition, our anticipated outcome was that this recognition and support from developing a network of colleagues would be present locally. Some participants indicated recognition within their department as well as from the school. Specifically, 12 respondents indicated they received a promotion and/or raise after completing the program.

“I was given a raise and title change. I am now the Program Manager for a new undergraduate program in the same department.” - *Pediatrics*

“I received a promotion and a raise as well as public acknowledgment from my Chair.” - *Internal Medicine*

“…very positively impacted [me] because of the networking with other administrators nationally and gaining an understanding of the various issues involved in clerkship administration. Having that broader perspective helped me understand where I am within my institution.” - *Pediatrics*

For some participants, their completion of the certificate program was not given any recognition locally.

“Until I received this survey, I forgot I even participated almost 10 years ago. If completing the project had been prioritized by my director, I think the program would have been more impactful.” - *Pediatrics*

“…was told by upper management that ‘anyone can go to a conference and get a certificate’. [They] totally disregarded the effort I put into the project ... It was truly heartbreaking.” - *Pediatrics*

## Discussion

The results of our survey indicated that the certificate program was a positive experience for all of the respondents. One of the goals of CACP was for clerkship administrators to invest in this role as a career. Completing the project was our proxy measure of them embracing this concept. Of the respondents, 72% completed the certificate program in its entirety and more than half went on to undertake more projects. Additionally, “goal orientation” from the realistic evaluation analysis indicated the programmatic intent was successful.

Applying the realistic evaluation framework to the survey data allowed us to better understand contextual issues faced by participants [6]. Results of CMO 1 (Germane) and CMO 2 (Applicable) were encouraging. The survey responses and narratives indicated that these aspects of the program are relatively successful. Participants indicated they applied leadership skills and communication skills after the workshops.

Project completion was mixed. Although many indicated confidence in undertaking a project, some faced barriers upon returning to their home institution. The project was initially promoted as more of an educational research project, which some participants noted their director was not interested in pursuing. We have now deliberately incorporated formal discussions at the end of the workshops to address how to go about doing a quality improvement project and how to collect information to demonstrate change. Additional resources are being added to the program website for additional guidance.

The mixed response for certificate completion highlights the importance of networking that began as part of the workshops. Respondents noted how impactful it was for them to meet others doing similar work. Offering follow-up networking sessions may promote developing a community of practice amongst the participants [11-12]. Facilitating additional informal sessions may enhance participants’ sense of agency to successfully complete a project [13].

Although it is made clear that completing this program is intended for personal growth, some participants indicated they were disappointed that they were not given more formal recognition locally for completing the certificate program. However, since many of the individuals completing the program attend conferences there could be a way to identify that they completed the certificate program on their name badges. For upcoming sessions, we will intentionally ask everyone why they are participating in the workshops. This will then allow us to further test our hypothesis that they feel local support for involvement in continuing professional development.

A limitation of this report is the lack of follow-up data regarding the long-term impact of this program on job satisfaction or continuing professional development of the participants. Although the impact has been noticeable through the presence of past participants who regularly attend ADMSEP, CGEA, or COMSEP meetings, the true magnitude has not been investigated. A future endeavor is to conduct follow-up studies with participants to inquire what sort of catalyst effect the certificate program has had on them. Additionally, it would be interesting to question clerkship directors for those who complete and do not complete the project requirement to better understand the goal orientation of participants. 

## Conclusions

Professional development opportunities for medical student education administrative staff is important. The impact of such programs has not been studied to date. Based on responses from participants of CACP, most completed the requirements of the program and more than half pursued additional projects. Using this data to make changes for future sessions, we will further explore the hypotheses generated from our realistic evaluation findings.

## Appendices

Supplemental Appendix:  Clerkship Administrator Certificate Program Survey

Q1 Do you still work in medical education in some capacity?  Yes/No

Q2 If NO, could you tell us why you left medical education for other opportunities? [Free text]

Q3 In what department did you work when you attended the Clerkship Administrator Certificate Program? [Free text]

Q4 What were your expectations attending the Clerkship Administrator Certificate Program? [Free text]

Q5 How were your expectations met (or not met) by participating? [Free text]

Q6 After attending the Clerkship Administrator Certificate Program, please indicate your level of agreement with the following statements:

I know what my values are in relation to my work. (1)

I recognize personality types better having completed the Myers-Briggs Test. (2)

I know the strengths I bring to my work. (3)

I recognize my areas for professional growth. (4)

I am confident in my leadership abilities and how they fit into my job. (5)

Scale used:  1=Strongly agree, 2=Agree, 3=Somewhat agree, 4=Neither agree/disagree, 5=Somewhat disagree, 6=Disagree, 7=Strongly disagree

Q7 How do you feel your career trajectory was impacted by your participation in the certificate program? [Free text]

Q8 Did you complete the project portion of the program?  Yes/No

Q9 If NO, what limitations or obstacles did you experience? [Free text]

Q10 If YES, please answer the following: Yes/No

Did you use your project in your clerkship/program/school?  (1)    

Did you undertake new projects?  (2)                                              

For new projects, did you submit them as presentations at other meetings?  (3)              

Did you attempt to publish your project? (4)                                    

Q11 Have you attended other regional or national medical education meetings? Yes/No

Q12 If NO, why?  If YES, how many and how often? [Free text]

Q13 How do you feel your personal life was impacted by the knowledge and skills learned in the program process? [Free text]
